# Enteric Pathogens and Reactive Arthritis: A Systematic Review of *Campylobacter*, *Salmonella* and *Shigella*-associated Reactive Arthritis

**DOI:** 10.3329/jhpn.v31i3.16515

**Published:** 2013-09

**Authors:** Anuli N. Ajene, Christa L. Fischer Walker, Robert E. Black

**Affiliations:** Department of International Health, Johns Hopkins Bloomberg School of Public Health, Baltimore, MD, USA

**Keywords:** *Campylobacter*, Enteric infections, Incidence, Reactive arthritis, *Salmonella*, *Shigella*

## Abstract

Reactive arthritis (ReA) is a spondyloarthropathic disorder characterized by inflammation of the joints and tissues occurring after gastrointestinal or genitourinary infections. Diagnostic criteria for ReA do not exist and, therefore, it is subject to clinical opinion resulting in cases with a wide range of symptoms and definitions. Using standardized diagnostic criteria, we conducted a systematic literature review to establish the global incidence of ReA for each of the three most commonly-associated enteric pathogens : *Campylobacter*, *Salmonella*, and *Shigella*. The weighted mean incidence of reactive arthritis was 9, 12, and 12 cases per 1,000 cases of *Campylobacter*, *Salmonella* and *Shigella* infections respectively. To our knowledge, this is the first systematic review of worldwide data that use well-defined criteria to characterize diarrhoea-associated ReA. This information will aid in determining the burden of disease and act as a planning tool for public-health programmes.

## INTRODUCTION

Reactive arthritis (ReA), also known as post-infectious arthritisis, is a spondyloarthropathic disorder characterized by inflammation of the joints and tissues occurring after gastrointestinal or genitourinary infections (1-3). The most common enteric bacterial pathogens that have been implicated in ReA include *Salmonella*, *Shigella*, and *Campylobacter* (4).

There is no diagnostic test for ReA; it is based entirely on clinical characteristics and, thus, is subject to clinical opinion (5). In addition, there are no established criteria for ReA diagnosis. Therefore, cases include a wide range of symptoms and definitions, some of which resemble other spondyloarthropathic disorders (1,5). A definite time period from infection to the onset of ReA has not been established which contributes to significant uncertainty and inconsistency between studies. This discordance has led to variability in incidence estimates for reactive arthritis (1,3).

Since there is considerable inconsistency in the diagnosis of ReA, it is important to determine the burden of reactive arthritis due to enteric infections, using standard criteria. To our knowledge, this is the first systematic review of worldwide data that use well-defined criteria to establish the global incidence of ReA for each of the three most commonly-associated pathogens.

## MATERIALS AND METHODS

We conducted a systematic literature review in Embase, PubMed, and Scopus databases, using all combinations of applicable MeSH and general terms for ReA, *Campylobacter*, *Salmonella*, and *Shigella* (Table 1a-1c). Additional articles were also retrieved from hand-searching reference lists of all articles selected for inclusion in the study. We sought to identify eligible case-control and prospective cohort studies that were published as of September 2011 in all languages. The inclusion criteria were laboratory confirmation of *Campylobacter*, *Salmonella*, or *Shigella* via stool or blood culture and ascertainment of ReA status within one year of infection. Prospective cohorts ascertained the clinical development of reactive arthritis via personal interview or chart reviews of individuals with laboratory-confirmed infections. For case-control studies, we searched for ones that tested for the presence of one of the selected ReA-triggering bacteria in both cases and controls. Cases had to be selected on the basis of ReA diagnosis with appropriate controls included.

**Table 1a-1c.** Database search terms

Table 1a	Embase	PubMed	Scopus
*Campylobacter*	‘reactive arthritis’/exp OR ‘reactive arthritis’ AND (‘campylobacter’/exp OR ‘campylobacter’) AND [humans]/lim AND [english]/lim	(“arthritis, reactive” [MeSH Terms] OR (“arthritis” [All Fields] AND “reactive” [All Fields]) OR “reactive arthritis” [All Fields] OR (“reactive” [All Fields] AND “arthritis” [All Fields])) AND (“campylobacter” [MeSH Terms] OR “campylobacter” [All Fields])	TITLE-ABS-KEY([40] OR {Reactive Arthritides} OR {Reactive Arthritis} OR {Post-Infectious Arthritis} OR {Post Infectious Arthritis} OR {Postinfectious Arthritis} OR {Arthritis Post-Infectious} OR {Arthritides Post-Infectious} OR {Arthritis Post Infectious} OR {Post-Infectious Arthritides} OR {Arthritis Postinfectious} OR {Arthritides Postinfectious} OR {Postinfectious Arthritides} OR {Reiter Syndrome} OR {Syndrome Reiter} OR {Reiter's Disease} OR {Disease Reiter's} OR {Reiters Disease} OR {Reiter Disease} OR {Disease Reiter}) AND TITLE-ABS-KEY ({campylobacter} OR {Campylobacter coli} OR {Campylobacter fetus} OR {Campylobacter hyointestinalis} OR {Campylobacter jejuni} OR {Campylobacter lari} OR {Campylobacter rectus} OR {Campylobacter sputorum} OR {Campylobacter upsaliensis}) AND (LIMIT-TO(LANGUAGE,”English”))
Table 1b	Embase	PubMed	Scopus
*Salmonella*	‘reactive arthritis’/exp OR ‘reactive arthritis’ AND (‘salmonella’/exp OR ‘salmonella’) AND [humans]/lim AND [english]/lim	(“arthritis, reactive” [MeSH Terms] OR (“arthritis” [All Fields] AND “reactive”[All Fields]) OR “reactive arthritis” [All Fields] OR (“reactive” [All Fields] AND “arthritis”[All Fields])) AND (“salmonella” [MeSH Terms] OR “salmonella” [All Fields])	TITLE-ABS-KEY({Arthritides Reactive} OR {Reactive Arthritides} OR {Reactive Arthritis} OR {Post-Infectious Arthritis} OR {Post Infectious Arthritis} OR {Postinfectious Arthritis} OR {Arthritis Post-Infectious} OR {Arthritides Post-Infectious} OR {Arthritis Post Infectious} OR {Post-Infectious Arthritides} OR {Arthritis Postinfectious} OR {Arthritides Postinfectious} OR {Postinfectious Arthritides} OR {Reiter Syndrome} OR {Syndrome Reiter} OR {Reiter's Disease} OR {Disease Reiter's} OR {Reiters Disease} OR {Reiter Disease} OR {Disease Reiter}) AND TITLE-ABS-KEY ({Salmonella} OR {Salmonella arizonae} OR {Salmonella enterica} OR {Salmonella enteritidis} OR {Salmonella paratyphi A} OR {Salmonella paratyphi B} OR {Salmonella paratyphi C} OR {Salmonella typhi} OR {Salmonella typhimurium}) AND (LIMIT-TO (LANGUAGE,”English”))
Table 1c	Embase	PubMed	Scopus
*Shigella*	‘reactive arthritis’/exp OR ‘reactive arthritis’ AND (‘shigella’/exp OR ‘shigella’) AND [humans]/lim AND [english]/lim	(“arthritis, reactive” [MeSH Terms] OR (“arthritis” [All Fields] AND “reactive” [All Fields]) OR “reactive arthritis” [All Fields] OR (“reactive” [All Fields] AND “arthritis” [All Fields])) AND (“shigella” [MeSH Terms] OR “shigella” [All Fields])	TITLE-ABS-KEY({Arthritides Reactive} OR {Reactive Arthritides} OR {Reactive Arthritis} OR {Post-Infectious Arthritis} OR {Post Infectious Arthritis} OR {Postinfectious Arthritis} OR {Arthritis Post-Infectious} OR {Arthritides Post-Infectious} OR {Arthritis Post Infectious} OR {Post-Infectious Arthritides} OR {Arthritis Postinfectious} OR {Arthritides Postinfectious} OR {Postinfectious Arthritides} OR {Reiter Syndrome} OR {Syndrome Reiter} OR {Reiter's Disease} OR {Disease Reiter's} OR {Reiters Disease} OR {Reiter Disease} OR {Disease Reiter}) AND TITLE-ABS-KEY ({shigella} OR {shigella boydii} OR {shigella dysenteriae} OR {shigella flexneri} OR {shigella sonnei} AND (LIMIT-TO(LANGUAGE,”English”))

We excluded studies that did not confirm the presence of gastrointestinal pathogens via laboratory methods, articles that did not specify the time lag between infection and the ascertainment of ReA, case reports, and review articles without original data. Articles with less than 15 subjects were also excluded. Full manuscripts were obtained for all eligible studies.

### Statistical methods

We fit the data to a logistic regression model (6) to calculate a weighted mean for the incidence of ReA associated with gastroenteritis (7).

## RESULTS

We identified 970 articles from Embase, PubMed, and Scopus databases that were screened for potential inclusion in the study (Figure). A total of thirty-six articles published between 1976 and 2010 met all inclusion criteria. These studies were conducted predominantly in Europe, with seven studies in North America (n=6) and Australia (n=1). The time to ascertainment of ReA or development of symptoms ranged from within a month to 1 year post-infection, and the sample-size for cohort studies ranged from 37 to 57,425. The results presented are based upon the pooled incidence rates only. Due to the lack of a standard definition of ReA, there was high heterogeneity between the studies (I-squared >95%); therefore, the meta-analysis model was not a good fit for the data. No case-control studies were identified; thus, it was not possible to calculate the background rate of ReA and the attributable rate of ReA due to enteric infections.

**Figure.**
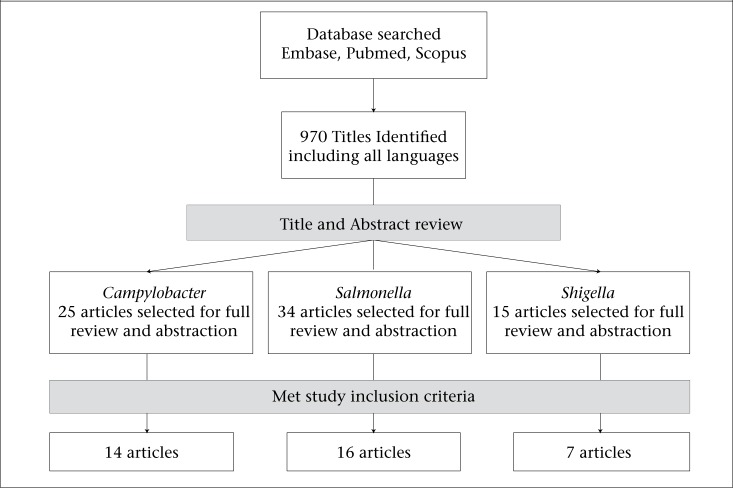
Literature review

### Campylobacter

We identified 25 articles for a full manuscript review. Out of these, 14 cohort studies (8-21) were included in the final assessment of the incidence of *Campylobacter-*associated reactive arthritis (Table 2). These cohort studies accounted for a total of 63,206 patients with *Campylobacter* infection, of which 573 progressed to reactive arthritis. The other 11 articles were excluded after reviewing the manuscripts due to either insufficient data on laboratory confirmation methods for all subjects, mixed bacterial isolates, information on time to ReA diagnosis missing or exceeding 12 months or they did not meet the other requirements for the study (22,23). The calculated pooled incidence rate was 0.009 (95% CI 0.009-0.010). This is an incidence of 9 reactive arthritis cases per 1,000 cases of *Campylobacter* infection.

**Table 2.** Cohort studies for *Campylobacter*

Author, Publication year	Country	Study duration	Population with *Campylobacter* infection	Population that developed ReA	Time from infection to ReA Diagnosis
Bremell, 1991	Sweden	1986-1987	66	5	<1 month
Eastmond, 1983	Scotland	1979	130	2	<2 months
Hannu, 2002	Finland	Apr 1997-Sept 1998	609	45	<2 months
Johnsen, 1983	Norway	Jun 1980-Sep 1981	37	5	3 days-2 weeks
Kosunen, 1981	Finland	Jul 1978-Dec 1979	342	8	4 days-4 weeks
Locht, 2002	Denmark	1997-1999	173	27	<4 weeks
Petersen, 1996	Denmark	Jan 1991-Dec 1993	41	0	NA
Pitkanen, 1983	Sweden	Jul 1978 -Jun 1981	188	9	1-3 week(s)
Ponka, 1984	Finland	Jul 1978-Jun 1981	283	6	2-3 weeks
Rees, 2004	United States	Apr 1998-Mar 1999	324	9	2 months
Schiellerup, 2008	Denmark	Jan 2002-Nov 2003	1,003	131	<4 weeks of infection
Schönberg-Norio, 2010	Finland	Jul 2002-Sep 2002	201	8	Median: 7 days (range:: 0-60 days)
Ternhag, 2008	Sweden	1997-2004	57,425	15	<12 months
Townes, 2008	United States	Mar 2002-Aug 2004	2384	302	Median/mean: 16/18 days; (Range: 0-180 days)

### Salmonella

We identified 34 articles for a full manuscript review. Out of these, 16 cohort studies (14,17,18,20,21,24-34) were included in the final assessment of the incidence of *Salmonella*-associated reactive arthritis (Table 3). These cohort studies accounted for a total of 39,148 patients with *Salmonella* infection, of which 474 progressed to reactive arthritis cases. Excluded articles did not contain relevant information or the time to ReA diagnosis was missing or exceeding 12 months, or data on laboratory confirmation methods for all subjects were insufficient. The calculated pooled incidence rate was 0.012 (95% CI 0.011-0.013). This is an incidence of 12 reactive arthritis cases per 1,000 cases of *Salmonella* infection.

### Shigella

We identified 15 articles for full manuscript review. Out of these, 7 cohort studies (14,17,18,20,21,35,36) were included in the final assessment of the incidence of *Shigella*-induced reactive arthritis (Table 4). These studies accounted for a total of 4,636 patients with *Shigella* infection, of which 56 reactive arthritis cases were found. The seven articles were excluded due to insufficient information either on laboratory confirmation for all subjects or ReA status, or data on time to ReA diagnosis was missing. The calculated pooled incidence rate was 0.012 (95% CI 0.009-0.015). This is an incidence of 12 reactive arthritis cases per 1,000 cases of *Shigella* infection.

## DISCUSSION

We sought to characterize the incidence of ReA among cases of *Campylobacter*, *Salmonella*, and *Shigella*, using well-established criteria. Among the studies analyzed, ReA incidence ranged from 0-16%, 0.1-29%, and 0-12% among *Campylobacter*, *Salmonella* and *Shigella* infections respectively. The weighted mean incidence was 9, 12, and 12 ReA cases per 1,000 *Campylobacter*, *Salmonella* and *Shigella* infections respectively.

It has been noted that gender does not play a role in enteric disease-associated ReA, and adults compared to children, are more susceptible to ReA following an enteric infection (3). In the present analysis, although only a small number of studies (Appendix) presented sufficient information to examine age-related ReA, we found a similar trend. The incidence of *Campylobacter*-associated ReA ranged from 8% to 16% with a median of 8% among adults compared to 0% to 6% with a median of 3% among children. *Salmonella-*associated ReA ranged from 1% to 24% with a median of 11% among adults compared to 0% to 12% with a median of 5% among children. *Shigella*-associated ReA ranged from 7% to 12% with a median of 9.5% among adults compared to 0% to 7% with a median of 3.5% among children. Although the burden of enteric infections is higher among children aged <5 years (37), we found a lower incidence of reactive arthritis in children which could be due to the lack of full differentiation of their immune systems (2,32). The information available on gender was insufficient to draw any conclusions.

**Table 3.** Cohort studies for *Salmonella*

Article	Country	Study duration	Population with *Salmonella i*nfection	Population that developed ReA	Time from infection to ReA diagnosis
Arnedo-Pena, 2010	Spain	Jul. 2005	67	6	Mean: 28 days (Range: 3-83 days)
Buxton, 2002	Canada	Dec 1999-Nov 2000	66	4	<3 months
Hakansson, 1976	Sweden	1974	330	13	1-2 weeks
Hannu, 2002	Finland	1999	63	5	Median: 2 days (Range 1-9 days)
Lee, 2005	Australia	Feb-Jun 1999	261	38	<3 months
Locht, 1993	Sweden	1990	113	17	<4 weeks
Mattila, 1994	Finland	1992	246	17	Median: 14 days (Range 7-40 days)
Mattila, 1998	Finland	1994	191	22	Median: 8.5 days (Range: 3-30 days)
Petersen, 1996	Denmark	Jan 1991-Dec 1993	135	8	During hospitalization, mean stay: 8 days (Range: 1-31 days)
Rees, 2004	United States	Apr 1998-Mar 1999	100	2	<2 months
Rudwaleit, 2001	Germany	1998	286	0	<4 months
Samuel, 1995	United States	Jul 1993	495	6	4-16 weeks
Schiellerup, 2008	Denmark	Jan 2002-Nov 2003	619	104	<4 weeks
Ternhag, 2008	Sweden	1997-2004	34,664	27	<12 months
Townes, 2008	United States	Mar 2002-Aug 2004	1,356	204	Median/mean: 16/18 days (Range: 0-180 days)
Urfer, 2000	Switzerland	Oct-Nov 1983	156	1	2 weeks

**Table 4.** Cohort studies for *Shigella*

Author	Country	Time of study	Population with *Shigella* infection	Population that developed ReA	Time from infection to ReA diagnosis
Hannu, 2005	Finland	Oct 1996-Sept 2000	14	211	<2 months
Lewis, 1982	United States	1980	0	127	NA
Petersen, 1996	Denmark	Jan 1991-Dec 1993	0	4	NA
Rees, 2004	United States	Apr 1998-Mar 1999	1	81	2 months
Schiellerup, 2008	Denmark	Jan 2002-Nov 2003	10	102	<4 weeks
Ternhag, 2008	Sweden	1997-2004	2	3,813	<12 months
Townes, 2008	United States	Mar 2002-Aug 2004	29	298	Median/mean: 16/18 days (Range: 0-180 days)

Our incidence estimates are comparable to previous estimates (3,18,32,35,38) but may under-estimate ReA cases for several reasons. ReA is considered to be a condition that may undergo spontaneous resolution. Therefore, individuals with milder cases of disease may not report it (39). In hospital-based studies, all patients are typically followed with either medical chart reviews or interviews with patients to assess the development of ReA, thereby minimizing loss to follow-up. This is in contrast to population-based studies where only a proportion of individuals with enteric infections may be assessed for ReA. For instance, such studies included in this review mailed out surveys to individuals with confirmed enteric infections to assess ReA symptoms or status, and only a proportion ranging from 31% to 90% (n=12) returned completed surveys. Incidence estimates were only based upon the sample that returned surveys which could bias our results in either direction if there was a differential response in those who developed ReA. We performed further sub-analyses, which excluded any study that had less than 70% of the subjects returning surveys (n=6), and the incidence estimates changed slightly with rates of 0.009 (95% CI 0.008-0.010) for *Campylobacter*, 0.011 (95% CI 0.010-0.012) for *Salmonella*, and 0.012 (95% CI 0.009-0.016) for *Shigella*.

Additionally, ReA is known to be a sequela of asymptomatic enteric infections (2,40). In this review, the ReA incidence estimates were based upon laboratory-confirmed cases of infection and may have missed those cases of ReA with negative laboratory findings or asymptomatic presentations, thereby leading to an under-estimation of the incidence. However, in the absence of a well-designed epidemiological study or population-wide screening, it would be difficult to ascertain the exact incidence of ReA in both symptomatic and asymptomatic cases.

Pope and colleagues (41) performed a review of *Campylobacter*-associated ReA and found an incidence of 1-5%. However, this review did not establish any inclusion or exclusion criteria, and a distinction was made between acute and chronic ReA. The authors noted that cases of ReA varied considerably due to limitations in case assessment and diagnostics for ReA. This review suggested that young adults were more commonly affected by ReA. Genetics, specifically the presence of human leukocyte antigen (HLA)-B27, was found to have a mixed association with ReA. There is considerable discussion regarding HLA-B27 and its role in ReA (1,42), and it is suggested that HLA-B27-positive individuals may have an increased susceptibility to ReA (43). However, the data on the association between HLA-B27 and ReA are conflicting with other studies finding no association (10,21).

We believe our review improves upon estimate of Pope and colleagues by imposing a timeframe for ReA case ascertainment, i.e. within a year of infection, and utilizing inclusion/exclusion criteria to increase uniformity among the estimates for the three enteric infections. These criteria assist in addressing the major limitation in estimating incidence for ReA, i.e. the lack of diagnostic criteria. Time to ascertainment of ReA was based upon the majority of studies that assessed ReA within one year post-enteric infection. Defining time to ReA can play an important role in the incidence estimates, for instance, one study that initially assessed ReA status among individuals with enteric infections found no cases within 3 months of infection. However, a re-assessment 1 year later in the same population discovered incident ReA cases (20). This indicates a potential for the number of ReA cases to be under-estimated, especially among studies with shorter timeframes.

We did not assess the role of treatment of enteric infections on the development of ReA as the data were sparse concerning the patients who received treatment. It has, however, been suggested that incidence of ReA is lower where prompt treatment is available (44). The data in this area are also limited and inconsistent with a randomized clinical trial (45) finding no association between antibiotic treatment and improved arthritis outcomes.

We present the incidence of ReA as sequelae of *Campylobacter*, *Salmonella* and *Shigella* infections, using worldwide data. We conducted a thorough search and used strict inclusion and exclusion criteria; yet, even with this method, we acknowledge that these estimates will have limitations until better diagnostic criteria become available. This includes stricter criteria for appropriate time intervals between infection and symptoms of ReA as well as standardized tests to detect bacterial antigens.

ReA remains an important consequence of gastrointestinal infections. These infections due to food-borne pathogens have been estimated to afflict up to 30% of the population in developed countries, with much higher attack rates in the developing world (46). With incidence rates between 1 and 2%, ReA affects a significant number of individuals worldwide, especially in countries that have high rates of food- and water-borne infections. Although ReA can be self-limiting, resolving within 6 months, it has been estimated that up to 63% of afflicted persons will develop a chronic form of ReA (3). This not only increases morbidity, it imposes an additional socioeconomic burden on individuals and societies. This highlights the need for early diagnosis of disease, prompt and effective treatment, and prevention of enteric infections. Improved estimates on the global incidence of ReA would serve as a tool in the development of appropriate public-health prevention programmes.

## ACKNOWLEDGEMENTS

The authors would like to thank Jamie Perin (JHSPH) for her assistance in statistical analysis.

**Appendix.** Age-related incidence of reactive arthritis

Bacteria	Author	Adult incidence of ReA (%)	Child incidence of ReA (%)
*Campylobacter*	Bremell, 1991	8	0
	Eastmond, 1983	8	6
	Hannu, 2002	16	-
	Johnsen, 1983	4	-
	Kosunen, 1981	15	-
*Salmonella*	Hannu, 2002	12	8
	Lee, 2005	9	0
	Locht, 1993	1	7
	Mattila, 1994	-	0
*Shigella*	Hannu, 2005	7	0
	Lewis, 1982	12	7
